# Psychological Mediators of the Association Between Childhood Emotional Abuse and Depression: A Systematic Review

**DOI:** 10.3389/fpsyt.2020.559213

**Published:** 2020-12-04

**Authors:** Elizabeth Tianyu Li, Patrick Luyten, Nick Midgley

**Affiliations:** ^1^Research Department of Clinical, Educational and Health Psychology, University College London, London, United Kingdom; ^2^Anna Freud National Centre for Children and Families, London, United Kingdom; ^3^Faculty of Psychology and Educational Sciences, University of Leuven, Leuven, Belgium; ^4^Child Study Center, School of Medicine, Yale University, New Haven, CT, United States

**Keywords:** depression, emotional abuse, child maltreatment, psychological mechanisms, mediator, early maladaptive schemas, cognitive-personality variables, emotion dysregulation

## Abstract

**Background:** A number of existing meta-analyses and narrative reviews have already addressed the relation between childhood adversity and depression, yet none of them has examined the specific link between emotional abuse and depression highlighted by previous research. It is no longer appropriate to regard childhood maltreatment as a unitary concept when considering its effects on subsequent depression; instead, subtypes of childhood maltreatment need to be scrutinized separately. This review addresses this significant gap by critically evaluating empirical studies examining psychological mediators of the relationship between childhood emotional abuse and subsequent depression.

**Methods:** A systematic search of nine electronic databases was conducted to identify eligible studies published in English between January 1980 and January 2020. Given the heterogeneous outcomes of eligible studies and the inconsistent reporting of indirect effects, a narrative synthesis, rather than a quantitative meta-analysis, was conducted. An appraisal of methodological quality was also included.

**Results:** We identified 34 papers, comprising 18,529 adults and 3,434 adolescents, including 888 clinical participants. Our synthesis suggests that studies on mediators in the emotional abuse–depression link have focused on five clusters of intervening variables: early maladaptive schemas, cognitive-personality variables, emotion dysregulation, interpersonal styles, and stressful negative events. Only 11 studies identified the unique contribution of emotional abuse to depression by controlling for other forms of childhood maltreatment.

**Conclusions:** Our findings support several routes with relative consistency (e.g., early maladaptive schemas, hopelessness, negative cognitive styles, brooding rumination, overall emotion dysregulation). Because psychological mediators function as a complex interrelated system, controlling for the interrelation between them is important. The evidence for the purported mediating role of the factors identified in this review should be considered with caution given the relative dearth of large-scale, adequately powered longitudinal studies. This review proposes a comprehensive multilevel theoretical framework as a basis for future research.

## Introduction

Both cross-sectional retrospective studies (1–3) and longitudinal studies (4–6) have provided evidence for the role of childhood maltreatment as a precursor to depression. Systematic reviews and meta-analyses (7–9) have consistently found childhood maltreatment to be strongly associated with clinical depression across the life course, an elevated risk of recurrent and persistent depressive episodes, and a lack of response or remission during treatment. In a recent meta-analysis of 184 studies totaling 255 effect sizes, maltreated individuals were found to be 2.66–3.73 times more likely to develop depression in adulthood, had an earlier onset of depression, and were twice more likely to develop chronic or treatment-resistant depression (10).

Whereas, the relation between childhood maltreatment and depression has been relatively well-established, much less is known about the relation between specific forms of maltreatment and depression. Historically, the more “obvious” forms of maltreatment, namely sexual and physical abuse, have attracted more attention when considering early adverse experiences associated with an increased risk of adolescent and adult depression (11, 12). Recently, however, researchers have started to examine particular types of childhood maltreatment as risk factors for depression, with an increasing focus on the more “silent” forms of maltreatment, such as emotional abuse. For example, studies have found that experience of parental physical abuse was least strongly associated with depressive symptoms (13), whereas self-reported childhood sexual abuse (14) and a history of emotional abuse have been more consistently associated with a wide range of psychopathology, and depression in particular (15–17). The term “childhood emotional abuse” has been used to describe exposure to spurning, terrorizing, isolating, exploiting or corrupting, and denying emotional responsiveness (18). Experiences of emotional abuse have been associated with powerful and enduring psychological sequelae, including shame, humiliation, anger, and feelings of worthlessness (19).

In a meta-analysis of 124 studies (20), emotional abuse was found to increase the risk of depression by an odds ratio of 3.06, whereas physical abuse increased the risk by an odds ratio of 1.5. In another meta-analysis of 184 studies (10), childhood emotional abuse was found to be most prominently linked to depression severity and was associated with the highest increase in the likelihood of depression in adulthood among all types of maltreatment. In addition, given that emotional abuse occurs in 90% of cases where the child is also physically or sexually abused and often precedes and persists beyond physical and sexual forms of abuse, it may be that the lasting psychological impact of different forms of abuse is largely explained by emotional abuse implicated in all forms of abuse (21). Although studies indicate that emotional abuse may play a key role in vulnerability for depression, emotional abuse is comparatively elusive, and its more “covert” nature often leads researchers and practitioners to focus on other more “tangible” forms of maltreatment in studying and treating depression (22, 23).

Relatively less is currently known about the potential psychological mechanisms linking emotional abuse and depression. Indeed, given that the time between exposure to abuse and onset of depression might be years or even decades, and that not all children who have experienced emotional abuse will go on to develop depression, the link between childhood emotional abuse and depression in adolescence and adulthood demands further explanation. Recent years have seen a vast increase in the number of studies investigating biological and psychosocial mechanisms addressing this issue (24). Biological mechanisms that have been investigated include genetic (25, 26) and neurobiological factors [e.g., impairments in HPA axis functioning; (27)]. Psychological mechanisms mainly involve maladaptive relationship and thinking styles, such as early maladaptive schemas (28), emotion dysregulation (29), hopelessness (30), insecure attachment styles (31), and interpersonal problems (32). Yet, the literature remains poorly integrated, and thus there is a need for a systematic review that has the potential to support the development of empirically supported models of depression vulnerability and maintenance and provides a platform for ensuring that psychological interventions target the most promising mechanisms, which may lead to improved treatment outcomes.

A number of existing meta-analyses and narrative reviews have already addressed the relation between adversity and depression, yet none of these meta-analyses focused on emotional abuse specifically. Braithwaite et al. (33) conducted a systematic review of longitudinal studies assessing the associations between childhood maltreatment in general and later depression. Interpersonal relationships (31, 34, 35), cognitive vulnerabilities (31, 36), and behavioral difficulties (37, 38) were found to mediate the relation between childhood maltreatment and depression. Hoppen and Chalder (24) conducted a systematic review of 214 studies on biopsychosocial mediating and moderating variables in the relationship between childhood adversity and affective disorders. They identified various psychological and social variables as mediators between childhood adversity in general and affective disorders in adulthood, including emotional dysregulation, attentional biases, psychoform dissociation, attachment anxiety, maladaptive cognitive styles, emotion-focused coping, lack of resilience, low self-esteem, trauma-related guilt, retraumatization, maladaptive personality types, anxious arousal, chronic interpersonal stress, and low social support. Fritz et al. (39) conducted a systematic review of social, emotional, cognitive, and behavioral resilience factors that may reduce the risk of psychopathology in young people following childhood adversity. They found empirical support for resilience factors at the individual (e.g., high self-esteem, low rumination), family (e.g., high family cohesion, high parental involvement), and community (i.e., high social support) level that may benefit mental health in young people who have experienced childhood adversity. Most recently, Aafjes–van Doorn et al. (40) specifically examined the mediating role of cognitive factors in the relationship between childhood trauma and subsequent adult psychopathology across clinical and non-clinical populations in a systematic review of 98 empirical studies. They found that cognitive factors consistently mediated this relationship, with the vast majority of studies reporting a significant mediation effect (95%; of which 83% was full mediation), regardless of different measures of traumatic experiences, psychopathology, and cognitive mediators. However, as noted, none of these reviews has examined the specific link between emotional abuse and depression highlighted by previous research (10, 20).

As a more hidden and “silent” form of childhood maltreatment, emotional abuse may be difficult to distinguish from dysfunctional parenting in general (41) and is often unrecognized and unrecorded by professionals (and wider systems) as well as laypersons (42). Regardless, empirical studies have suggested that the incidence of emotional abuse in the absence of other forms of maltreatment is more common than often assumed (43, 44). In the U.S. National Child Traumatic Stress Network (NCTSN) Core Data Set (CDS), emotional abuse and neglect were found to be the most prevalent form of maltreatment, where nearly one quarter (24%) of maltreatment cases exclusively involved emotional maltreatment (45, 46). Not only is it the case that different forms of child maltreatment were reported to have equivalent psychiatric effects (47), but the occurrence of emotional abuse was found to be an equivalent or significantly greater predictor of 27 out of 30 negative outcomes compared with the co-occurrence of physical and sexual abuse (43). All of this points to the role that emotional abuse may play as a powerful form of childhood abuse in its own right. Given that emotional abuse constitutes a distinct form of maltreatment; misattributing its pernicious effects to other more “tangible” forms of maltreatment also has important implications for clinical practice (48). It is no longer appropriate to regard childhood maltreatment as a unitary construct; instead, subtypes of childhood maltreatment need to be scrutinized separately as well as in combination.

The current review aims to address this significant gap in the evidence base and provides a systematic review and synthesis of the empirical literature for the proposed psychological mechanisms of the relationship between childhood emotional abuse and subsequent depression, taking into consideration the robustness of the statistical mediation methods employed. Specifically, this review aims to (a) provide a comprehensive systematic review of quantitative studies investigating psychological mediators between childhood emotional abuse and depression in adolescence and in adulthood, and (b) evaluate the quality of the available evidence, including the relative strength of the statistical mediation analysis used to explain the emotional abuse–depression link.

## Methods

### Search Procedure

The current review was designed in accordance with the Preferred Reporting Items for Systematic Reviews and Meta-analyses (PRISMA) guidelines (49, 50). Details of the protocol, including methods of the analyses and inclusion criteria, were specified in advance and documented on PROSPERO (PROSPERO registration: CRD42019127975). A systematic literature search was conducted twice by the first author, the first time in February 2019 when registering the study in PROSPERO, the second time in January 2020 to assure that more recent studies were also included. These two systematic searches identified the same set of 30 empirical studies to be included in this review and four additional studies that were published since the first review (51–54). Nine electronic databases—PsycINFO, Web of Science, MEDLINE, Scopus, and ProQuest (including PTSDpubs database, Health & Medical Collection, Research Library, Science Database, and Social Science Database)—were systematically searched using the following search terms: (depress OR internalizing OR Affective Disorder OR Mood Disorder) AND (emotional abuse OR emotional maltreatment OR verbal abuse OR emotional trauma OR psychological abuse OR child abuse OR child maltreatment). The search terms followed closely those of previously published meta-analyses of the relationship between childhood emotional abuse and depression. As different terms are used to describe mediators or mechanisms in different disciplines, we did not restrict our search to terms relating to psychological mediators. Duplicates were filtered out using EndNote Web. Examination of reference lists of eligible studies and a forward search was carried out in addition to the database search.

### Inclusion and Exclusion Criteria

Eligible studies were original empirical studies that investigated the relationship between childhood emotional abuse and depression and published between 1980 and 2020 in peer-reviewed journals. Eligible designs were cross-sectional, case-control, and prospective cohort (over any time period) studies that had assessed the association between childhood emotional abuse and depression symptoms and/or diagnosis of depression and the effect of one or more psychological mediating mechanisms on this relationship. Both a diagnosis of clinical depression and a continuous measure of depressive symptoms using scales with reported validity and reliability were considered eligible because there is evidence that depression can exist on a continuum of severity, ranging from mild, transient depressed mood states to severe, chronic forms.

Only reports that utilized mediation analysis or another suitable modeling approach to examine whether childhood emotional abuse had an indirect effect on depression outcomes via specific psychological processes were included. Our definition of psychological mechanisms was adapted from that of Harvey and Watkins (55) and considered any aspect of cognition, behavior, affective symptoms, or mood. Studies were regarded as eligible if the measures used to assess the variables under study (emotional abuse, symptoms/diagnoses, and putative mediating mechanisms) were described as valid and reliable. However, where studies were epidemiological in nature, measures of variables using items created for the study without further additional validation were considered eligible given that population-based studies often employ extensive surveys, necessitating briefer means of assessing each variable of interest.

Participants were adolescents or adults from any background and in any setting, including inpatients. Studies that examined the mediating mechanisms between parental emotional abuse and current depression in a child sample or emotional abuse perpetrated by peers were excluded (56). Studies that used a single item from a validated scale were also excluded, as were studies that calculated the overall effect of childhood maltreatment only and not emotional abuse specifically (57–61). Studies in languages other than English were excluded, as were single-case studies, case series, qualitative studies without a quantitative element, review articles, book chapters, dissertations, discussion papers, non-research letters, editorials, and conference abstracts.

### Data Extraction and Analytic Plan

After the removal of duplicates, paper titles were analyzed by the first author. The majority of articles excluded at this stage had titles referring to either completely unrelated topics or did not meet the inclusion and exclusion criteria listed above. The abstracts of the remaining articles were then screened by the first author. All papers potentially meeting inclusion and exclusion criteria were included for full-text scrutiny. Uncertainties concerning whether a study should be included or excluded were resolved through discussions between the authors. To ensure that relevant papers were not missed, additional screening measures, consisting of conducting a citation search and screening the references of eligible studies, were conducted. The reference lists were manually checked for studies not retrieved via electronic searches.

Information about study design, sample, measures of variables, the statistical analysis used to test mediation, and outcomes of interest was extracted and integrated by using a narrative integration approach structured around the type of mediator examined (see Table 1). Given the heterogeneous outcomes of eligible studies (i.e., in terms of psychological mediators, adolescent and adult depression symptoms or diagnoses) and the inconsistent reporting of indirect effects, a narrative synthesis, rather than a quantitative meta-analysis, was conducted. Some studies did report model-related fit indices for mediation (e.g., Root Mean Square Error of Approximation) but the majority of the studies did not report mediator-related effect sizes. Hence, statistical comparison of effect sizes was not feasible.

**Table 1:** Overview of studies included in review.

**References; Country**	**Design**	**Sample**	**Measures Childhood emotional abuse**	**Measures Mediator(s)**	**Measures Depression**	**Analysis of mediation**	**Main relevant findings**
Calvete (62); Spain	Longitudinal	1,052 adolescents aged 13–17 years (499 girls, 553 boys, mean age = 13.43, *SD* = 1.29)	The 5-item Psychological Abuse Scale of the CTS-PC	YSQ-3	CES-D	Structural equation modeling, autoregressive model, bootstrapping procedure (177)	Parental emotional abuse did not predict the worsening of early maladaptive schemas in the follow-ups but was directly associated with depressive symptoms
Carvalho Fernando et al. (63); Germany	Cross-sectional	160 adults (49 BPD patients, 48 MDD patients, 63 controls)	CTQ	DERS, ERQ	BDI	Multiple regression analyses	No significant impact of self-reported childhood emotional abuse and emotion dysregulation on depressive symptoms was found
Christ et al. (32); The Netherlands	Cross-sectional	276 female college students with a mean age of 21.7 years (*SD* = 2.38)	CTQ-SF	DERS, IIP-32	Quick Inventory of Depressive Symptoms	Multiple regression analyses, the PROCESS tool (88)	The effect of childhood emotional abuse on depressive symptoms was mediated by emotion dysregulation and the following domains of interpersonal problems: cold/distant and domineering/controlling
Coates and Messman-Moore (64); USA	Cross-sectional	771 female undergraduate students between the ages of 18 and 25 with a mean age of 18.78 (*SD* = 1.02)	The Computer Assisted Maltreatment Inventory	DERS, YSQ-SF	TSI	Structural equation modeling	Both emotion dysregulation and negative internalized beliefs significantly mediated the link between childhood psychological maltreatment and depressive symptoms, accounting for ~68% of the variance in symptomatology
Courtney et al. (65); USA	Cross-sectional	195 adolescent primary care patients (21.5% male, 78.5% female), aged 15–18 years (*M* = 16.30, *SD* = 1.07)	Three items adapted from CTQ that directly assess verbal and nonverbal emotional abuse	BHS	BDI	Multiple regression analyses	Hopelessness partially mediated the associations of emotional abuse with risk for depressive symptoms. Hopelessness accounted for 69.8% of the variance in the association between emotional abuse and depressive symptoms
Courtney et al. (30); USA	Longitudinal	92 adolescent primary care patients (18.5% boys, 81.5% girls) between 15 and 18 years of age (*M* = 17.48, *SD* = 1.27)	Three items adapted from CTQ that directly assess verbal and nonverbal emotional abuse	BHS	BDI-II	Multiple regression analyses	Hopelessness partially mediated the association between T1 emotional abuse and T2 depressive symptoms. Hopelessness accounted for 87.3% of the variance in this association
Crow et al. (29); USA	Cross-sectional	3,902 adults aged 18–81 years (*M* = 39.34, *SD* = 13.76, 68.9% women, 92.7% African American)	CTQ	EDS	BDI-II	(73)	Emotion dysregulation mediated the relationship between childhood emotional abuse and later depression
Gibb et al. (66); USA	Longitudinal	297 university students (145 high cognitive risk, mean age = 18.92, 68.3% female; 152 low cognitive risk, mean age = 19.28, 68.4% female)	The maltreatment subscale of the LEQ	CSQ, DAS	BDI, BHS, SADS	Hierarchical regression analysis	Cognitive risk fully mediated the relation between reported levels of childhood emotional maltreatment and non-endogenous major depression as well as hopelessness depression
Gibb et al. (67); USA	Cross-sectional	220 undergraduates aged 17–26 years (164 women, 56 men, mean age = 18.79, *SD* = 1.40)	LEQ	BHS	HDSQ	Path analyses using AMOS 4.0	Hopelessness partially mediated the relation between childhood emotional maltreatment and symptoms of hopelessness depression
Hankin (31); USA	Study 1: Longitudinal (10 weeks)	Study 1: 652 undergraduate students aged 17–23 years (mean = 18.7, *SD* = 0.96, 210 males)	Study 1: LEQ	Study 1: CSQ, NLEQ, AAQ	Study 1: BDI, MASQ	Study 1: Path analyses (68), univariate and multivariate mediational analyses (178)	Study 1: An insecure attachment style and negative life events almost completely mediated the association between childhood emotional abuse and later depressive symptoms, while a negative cognitive style was minimized in the multivariate mediational model
	Study 2: Longitudinal (2 years)	Study 2: 75 undergraduate students aged 18–23 years (mean = 18.6, *SD* = 0.84, 34 males)	Study 2: CECA	Study 2: CSQ, NLEQ, AAQ	Study 2: BDI, MASQ	Study 2: Path analyses (68), univariate and multivariate mediational analyses (178)	Study 2: Only an insecure attachment style and negative life events remained partially accounted for the association between childhood emotional abuse and later depressive symptoms in the multivariate mediational model
Hayashi et al. (69); Japan	Cross-sectional	113 untreated, newly diagnosed MDD patients aged 25–75 years (58 women and 55 men, mean age 41.91 years, *SD* = 11.20)	CATS	LES, NEO-FFI	BDI-II	Structural equation modeling	Childhood emotional abuse predicted the severity of depression indirectly through the mediation of personality: Neuroticism, Extroversion, and Conscientiousness. The negative life change was affected by childhood emotional abuse but did not predict the severity of depression
Jessar et al. (70); USA	Longitudinal	204 early adolescents (52% African American, 54% female, mean age = 12.85 years)	The emotional abuse and emotional neglect subscales of the CTQ	ECQ	CDI	(73)	Emotional abuse did not significantly predict deficits in emotional clarity but did predict increases in depressive symptoms. Deficits in emotional clarity only mediated the relationship between emotional neglect and increases in depressive symptoms
Kaysen et al. (71); USA	Cross-sectional	206 adult women who had been recently raped (*N* = 133) or physically assaulted (*N* = 73) (mean age = 31.21 years, *SD* = 8.58, range = 18–57)	HVQ	PBRS	BDI, SCID-MDD	(68)	No evidence for associations of childhood emotional abuse with either maladaptive cognitions or depression
Khosravani et al. (52); Iran	Cross-sectional	350 males (age range = 18–61 years, *M* = 32.3)	CTQ-SF	DERS	BDI-II	Structural equation models (SEM), bootstrapping	A direct effect of emotional abuse on depressive symptoms and an indirect effect via emotion dysregulation
Krause et al. (72); USA	Cross-sectional	127 adults aged 18–30 years (mean age = 20, *SD* = 2.76, 78 women, 76% Caucasian)	Psychological Abuse Scale (179)	WBSI, AEQ, CSQ, the 11-item avoidant reactions subscale of the Impact of Event Scale	BDI	Structural equation modeling, AMOS	Chronic emotional inhibition (including ambivalent expression, thought suppress, current and chronic avoid) fully mediated the relationship between childhood emotional invalidation (including emotional abuse) and depressive symptoms in adulthood
Li et al. (54); UK	Cross-sectional	205 adults (80.5% female; mean age = 28.2, *SD* = 10.86)	7-item Childhood Emotional Abuse Scale created and validated by Kent and Waller (180)	RFQ	7 items of the Depression subscale in the DASS-21	Multiple regression analyses, PROCESS macro (88)	Emotional abuse continued to exert a significant effect on adulthood depression after controlling for other forms of childhood maltreatment and mentalizing incapacity. A mediation effect between childhood emotional abuse and adulthood depression symptoms via mentalizing incapacity (both hypermentalizing and hypomentalizing) was established
Lumley and Harkness (28); Canada	Cross-sectional	76 depressed adolescents meeting DSM-IV criteria for a current episode of a non-bipolar mood disorder, aged 13–19 years (24 boys, 52 girls)	The teenage version of the CECA	YSQ	MASQ, BDI-II, The child and adolescent version of the SADS	(68, 73)	A different set of early maladaptive schemas with contents, such as social isolation and self-sacrifice, mediated the relation of emotional maltreatment to anhedonic depression. But no specificity was found in the relation between emotional maltreatment and anhedonic depression
Lumley and Harkness (28); Canada	Cross-sectional	91 young adults aged 17–21 years (21 men, 70 women, mean age = 18.10, *SD* = 0.84)	CECA.Q	YSQ, The Modified-Psychological Distance Scaling Task	BDI, the Mood Disorder Module of the SCID	(68), Sobel test	Depressotypic cognitive organization (i.e., tightly connected negative schema organization and loosely connected positive schema organization) mediated the relation between childhood emotional maltreatment and young adult depression
Maciejewski and Mazure (74); USA	Case-control	50 adults (25 cases, 25 controls) aged 23–65 years (*M* = 39.6, *SD* = 10.3, 24 women, 42 European American descent)	ETI	The Fear of Criticism and Rejection subscale in Beck's Sociotropy-Autonomy Scale	SCID, CES-D, HRSD, the University of Michigan version of CIDI	Multiple logistic regression, multivariate analysis of covariance (MANCOVA)	Fear of criticism and rejection mediated the association between childhood emotional abuse and adult onset of major depression
O'Mahen et al. (21); USA	Cross-sectional	140 pregnant women, age 18 or older, 24 or more weeks pregnant	CTQ	RRS, BADS	SCID-I, EPDS, BDI-II	Nested path models in AMOS 18.0, bootstrapping procedure (177)	Brooding partially mediated the relationship between childhood emotional abuse and depressive symptoms, whereas behavioral avoidance was not significantly correlated with childhood emotional abuse
Østefjells et al. (75); Norway	Cross-sectional	261 patients aged 18–65 years with psychotic or bipolar disorders	CTQ-SF	The subscale measuring negative beliefs about the uncontrollability and danger of thoughts of the Metacognitions Questionnaire-30 items	Positive and Negative Syndrome Scale Score	Ordinary least-squares regressions with the PROCESS tool (88)	Metacognitive beliefs about thoughts being uncontrollable/dangerous significantly mediated the relationship between early emotional abuse and depression
Paredes and Calvete (36); Spain	Longitudinal	998 adolescents (471 girls, 526 boys) between 13 and 17 years of age	The 6-item version of the Emotional Abuse Scale adapted from CTS-PC	CRSS, ACSQ	CES-D	Structural equation modeling, Sobel test	Only brooding partially mediated the relationship between emotional abuse by parents and depressive symptoms. Neither reflection nor negative inferential styles increased vulnerability to depression
Raes and Hermans (76); Belgium	Cross-sectional	101 students (83 women, mean age = 19.64 years)	The emotional abuse subscale of the CTQ	RRS	BDI	Multiple regression analyses, Sobel test	Brooding partially mediated the relationship between emotional abuse and depression, even when reflection was partialed out
Rafi et al. (77); Iran	Cross-sectional	492 middle school students (183 boys, mean age = 13.61 years, *SD* = 0.682, 309 girls mean age = 13.60, *SD* = 0.572)	Child Abuse Self Report Scale	SIC	The anxiety and depression subscales of the ASEBA	Path analysis	Early maladaptive schemas (i.e., loneliness, vulnerability to harm, and submission) mediated the relationship between childhood emotional maltreatment and depression
Reddy et al. (78); USA	Cross-sectional	987 college undergraduates (52.5% males, 65% Caucasian, 93% below 22 years old)	FEQ	EAS, WBSI, Acceptance and Action Questionnaire	DASS-21	Structural equation modeling	Experiential avoidance (examined by three measures) significantly mediated the relationship between childhood psychological abuse and current mental health symptoms (including depression, anxiety and stress), reducing the direct effect by 77%
Ross et al. (53); USA	Cross-sectional	244 adults (53 males; mean age = 20.80, *SD* = 3.826)	CTQ	ISS, the 26-item Self-Compassion Scale	CESD-R	Path models, bootstrapping	A significant path from emotional abuse to depression, and a significant indirect path that passed through self-compassion and shame
Sachs-Ericsson et al. (79); USA	Cross-sectional	5614 adults age range 15–54 years (*M* = 33.2 years, *SD* = 10.7)	A list of specific behaviors related to parental verbal abuse, including insulted, swore at, did or said something to spite, threatened to hit.	4 items of self-criticism from the DEQ, Dependency and Emotional Reliance on Others Scale (181)	CIDI	Hierarchical linear regression analyses, Sobel test	Self-criticism fully mediated the relationship between parental verbal abuse and internalizing symptoms (including symptoms of depression), whereas dependency did not mediate the relationship between any abuse and internalizing symptoms
Schulz et al. (80); Germany	Longitudinal	123 inpatients aged 18–65 years with current MDD diagnosis	The emotional abuse and emotional neglect subscales of the CTQ	PSDI, EAQ	BDI-II, Montgomery–Åsberg Depression Rating Scale	(73), Sobel test	Borderline personality traits and acceptance of pleasant emotions are significant mediators of the association between childhood emotional abuse and self-rated depression severity. Childhood emotional abuse is not correlated to expert-ratings of depression
Spasojevic and Alloy (81); USA	Longitudinal	137 undergraduate students aged 16–29 years (88 females and 49 males, mean age = 19)	LEQ	RRS	BDI, Mod-SADS-L	Hierarchical regression analysis (68)	The relationship between childhood emotional maltreatment and the number of major depressive episodes was partially mediated by ruminative response style
Uhrlass and Gibb (82); USA	Longitudinal	208 undergraduate students (mean age = 19.6 years, *SD* = 4.3, 148 females, 58.2% Caucasian)	LEQ	The 53-item Hassles subscale of the Hassles and Uplifts Scale	BDI-II	Path analysis, AMOS 5.0	Changes in recent negative events fully mediated, rather than moderated, the link between reports of childhood emotional maltreatment and changes in depressive symptoms
Van Assche et al. (51); Belgium	Cross-sectional	81 older adults age range 62–90 (36% males; *M* = 74.90, *SD* = 6.64)	CTQ-SF	ECR-R	GDS	Bootstrapping	Childhood emotional abuse was not significantly correlated with current depression or attachment. Both attachment anxiety and attachment avoidance showed a significant positive correlation with the current level of depression
van Harmelen et al. (83); The Netherlands	Cross-sectional	2837 adults aged between 18 and 65 years (66.5% female, age *M* = 41.9 years, *SD* = 13.0)	The Netherlands Mental Health Survey and Incidence Study (NEMESIS) trauma interview	Implicit Association Test	CIDI, IDS	(73)	Both automatic and explicit negative self-associations partially mediated the relationship between childhood emotional maltreatment and depressive symptomatology
Wright et al. (84); USA	Cross-sectional	301 undergraduate students (143 men, 158 women, mean age = 20.37, 94.4% Caucasian)	LEQ	YSQ	TSC-40	Hierarchical regression analysis (68)	The schemas of vulnerability to harm, self-sacrifice, and defectiveness/shame mediated the relationship between childhood emotional abuse and adult symptoms of depression
Yigit et al. (85); Turkey	Cross-sectional	325 participants in total (13–18 years old): 193 clinical adolescents (129 girls; mean age = 15.65, *SD* = 1.15); 132 non-clinical adolescents (94 girls; mean age = 15.05, *SD* = 1.07)	CTQ	YSQ-3	CDI	Path analysis, bootstrapping	Disconnection/Rejection and Impaired Autonomy mediated significantly emotional abuse and depression in the non-clinical sample. Disconnection/Rejection significantly mediated the relationship between emotional abuse and depression in the clinical sample

*CTQ, Childhood Trauma Questionnaire; CTQ-SF, Childhood Trauma Questionnaire-Short Form; BDI, Beck Depression Inventory; BDI-II, Beck Depression Inventory II; YSQ, Young Schema Questionnaire; YSQ-SF, Young Schema Questionnaire–Short Form; YSQ-3, Young Schema Questionnaire-3; CECA, Childhood Experience of care and Abuse; CECA.Q, Childhood Experience of Care and Abuse questionnaire; HADS, Hospital Anxiety and Depression Scale; ECR-R, Experiences in Close Relationships Questionnaire-Revised; CATS, Child Abuse and Trauma Scale; SCID, Structured Clinical Interview for DSM-IV; SCID-I, Structured Clinical Interview for DSM-IV Axis 1 Disorders-Patient Edition; SCID-MDD, Structured Clinical Interview for DSM-III-R Non-Patient Version; DERS, Difficulties in Emotion Regulation Scale; ERQ, Emotion Regulation Questionnaire; IIP-32, Inventory of Interpersonal Problems; CSQ, Cognitive Style Questionnaire; DAS, Dysfunctional Attitude Scale; TSI, Trauma Symptom Inventory; BHS, Beck Hopelessness Scale; EDS, Emotion Dysregulation Scale; LEQ, Lifetime Experiences Questionnaire; NLEQ, Negative Life Events Questionnaire; LES, Life Experiences Survey; NEO-FFI, Neuroticism Extroversion Openness Five Factor Inventory; ECQ, Emotional Clarity Questionnaire; DASS-21, Depression Anxiety Stress Scale−21item; MASQ, Mood and Anxiety Symptom Questionnaire; WBSI, White Bear Thought Suppression Inventory; AEQ, Ambivalence Over Emotional Expressiveness Questionnaire; HVQ, History of Victimization Questionnaire; RFQ, Reflective Functioning Questionnaire; CDI, Children's Depression Inventory; CES-D, Center for Epidemiological Studies Depression Scale; CESD-R, Center for Epidemiological Studies Depression Scale-Revised; SADS, Schedule for Affective Disorders and Schizophrenia; Mod-SADS-L, A modified Schedule for Affective Disorders and Schizophrenia – Lifetime; HDSQ, Hopelessness Depression Symptom Questionnaire; ETI, Early Trauma Inventory; RRS, Ruminative Responses Scale; BADS, Behavioral Activation for Depression Scale; EPDS, Edinburgh Postnatal Depression Screen; SIC, A Schema Inventory for Children; ASEBA, Achenbach System of Empirically Based Assessment; FEQ, Family Experiences Questionnaire; ISS, Internalized Shame Scale; EAS, Experiential Avoidance Scale; PBRS, Personal Beliefs and Reactions Scale; GDS, Geriatric Depression Scale; EAQ, Emotion Acceptance Questionnaire; PSDI, Personality Style and Disorder Inventory; DEQ, Depressive Experiences Questionnaire; HRSD, Hamilton Rating Scale for Depression; ACSQ, Adolescent Cognitive Style Questionnaire; CRSS, Children's Response Style Scale; CTS-PC, Conflict Tactics Scales-Parent-to-Child; TSC-40, Trauma Symptom Checklist-40; IDS, Inventory of Depressive Symptomatology; AAQ, Adult Attachment Questionnaire; CIDI, Composite International Diagnostic Interview*.

In the present review, we grouped studies according to five types of psychological mediators in the emotional abuse–depression link: these were early maladaptive schemas, cognitive-personality variables, emotion dysregulation, interpersonal styles, and stressful negative events. These five types of mediating variables are neither necessarily independent nor mutually exclusive, but we considered them separately. In the Discussion we discuss to what extent these potential mediators might be hierarchically organized.

### Quality Assessment

The first author independently evaluated study quality. Uncertainties concerning study quality were resolved through discussions between the authors. Following methodological recommendations from PRISMA (49), a component approach to quality assessment was employed. The quality of eligible papers was assessed using the Effective Public Health Practice Project tool [EPHPP; (86)] adapted to enable assessment of the specific methodological features of the primary studies pertinent to the research question under scrutiny. Our quality assessment considered the following four domains: (a) selection bias, (b) study design, (c) confounders, (d) data collection methods, and (e) withdrawals and dropouts. The analytic approach employed was also assessed and rated as “strong,” “moderate,” or “weak” depending on its appropriateness in terms of testing for mediation effects. Regression methods (68) where mediational effects are inferred rather than based on direct statistical observation (87) were rated as weak. A moderate rating was assigned to analyses where regression methods with additional tests of indirect effects, such as the Sobel test, had been used in addition to regression analysis. Explicit analyses estimating direct and indirect effects with bootstrapping techniques (73, 88) and path analysis were assigned a strong rating. Table 2 presents a summary of the quality of each eligible study.

**Table 2:** Quality assessment tool ratings.

**References; Country**	**Selection bias**	**Study design**	**Confounders**	**Data collection methods**			**Withdrawals and dropouts**	**Mediation analyses**
				**IV**	**M**	**DV**		
Calvete (62); Spain	W	M	W	S	S	S	M	S
Carvalho Fernando et al. (63); Germany	M	W	S	S	S	S	M	W
Christ et al. (32); The Netherlands	W	W	S	S	Emotion dysregulation = S Interpersonal problems = S	S	M	S
Coates and Messman-Moore (64); USA	W	W	W	S	Emotion dysregulation = S Negative internalized beliefs = S	S	M	S
Courtney et al. (65); USA	M	W	W	S	S	S	M	W
Courtney et al. (30); USA	M	M	M	S	S	S	W	W
Crow et al. (29); USA	M	W	S	S	M	S	M	S
Gibb et al. (66); USA	W	M	M	S	S	S	S	W
Gibb et al. (67); USA	W	W	W	S	S	S	M	S
Hankin (31); USA Study 1 Study 2	W W	M S	S S	S S	Insecure attachment style = S Negative cognitive style = S Negative events = S	S S	S S	S S
Hayashi et al. (69); Japan	M	W	W	S	Personality = S Stress of life events = S	S	M	S
Jessar et al. (70); USA	W	M	M	S	M	S	W	S
Kaysen et al. (71); USA	M	W	W	W	S	S	M	W
Khosravani et al. (52); Iran	M	W	S	S	S	S	M	S
Krause et al. (72); USA	W	W	W	W	S	S	M	S
Li et al. (54); UK	W	W	W	S	S	S	M	S
Lumley and Harkness (28); Canada	M	W	M	S	S	S	M	S
Lumley and Harkness (89); Canada	W	W	M	S	S	S	M	M
Maciejewski and Mazure (74); USA	M	M	S	S	S	S	M	W
O'Mahen et al. (21); USA	M	W	M	S	Brooding = S Behavioral avoidance = S	S	M	S
Østefjells et al. (75); Norway	M	W	M	S	S	S	M	S
Paredes and Calvete (36); Spain	W	M	W	S	Brooding = S Negative inferential styles = S	S	M	S
Raes and Hermans (76); Belgium	W	W	W	S	S	S	M	M
Rafi et al. (77); Iran	W	W	W	S	W	W	M	S
Reddy et al. (78); USA	W	W	S	W	S	S	M	S
Ross et al. (53); USA	W	W	W	S	Self-compassion = S Shame = S	S	M	S
Sachs-Ericsson et al. (79); USA	S	W	S	W	Self-criticism = W Dependency = W	S	M	M
Schulz et al. (80); Germany	M	M	S	S	Borderline personality = S Low acceptance of pleasant emotions = W	S	W	S
Spasojevic and Alloy (81); USA	W	M	M	S	S	S	W	W
Uhrlass and Gibb (82); USA	W	M	W	S	S	S	W	S
Van Assche et al. (51); Belgium	M	W	S	S	S	S	M	S
van Harmelen et al. (83); The Netherlands	S	W	S	S	S	S	M	S
Wright et al. (84); USA	W	W	S	S	S	S	M	W
Yigit et al. (85); Turkey	M	W	M	S	S	S	M	S

*S, strong; M, moderate; W, weak; IV, independent variable, M, mediator(s); DV, dependent variable*.

## Results

### Characteristics of the Primary Studies

**Figure 1:**
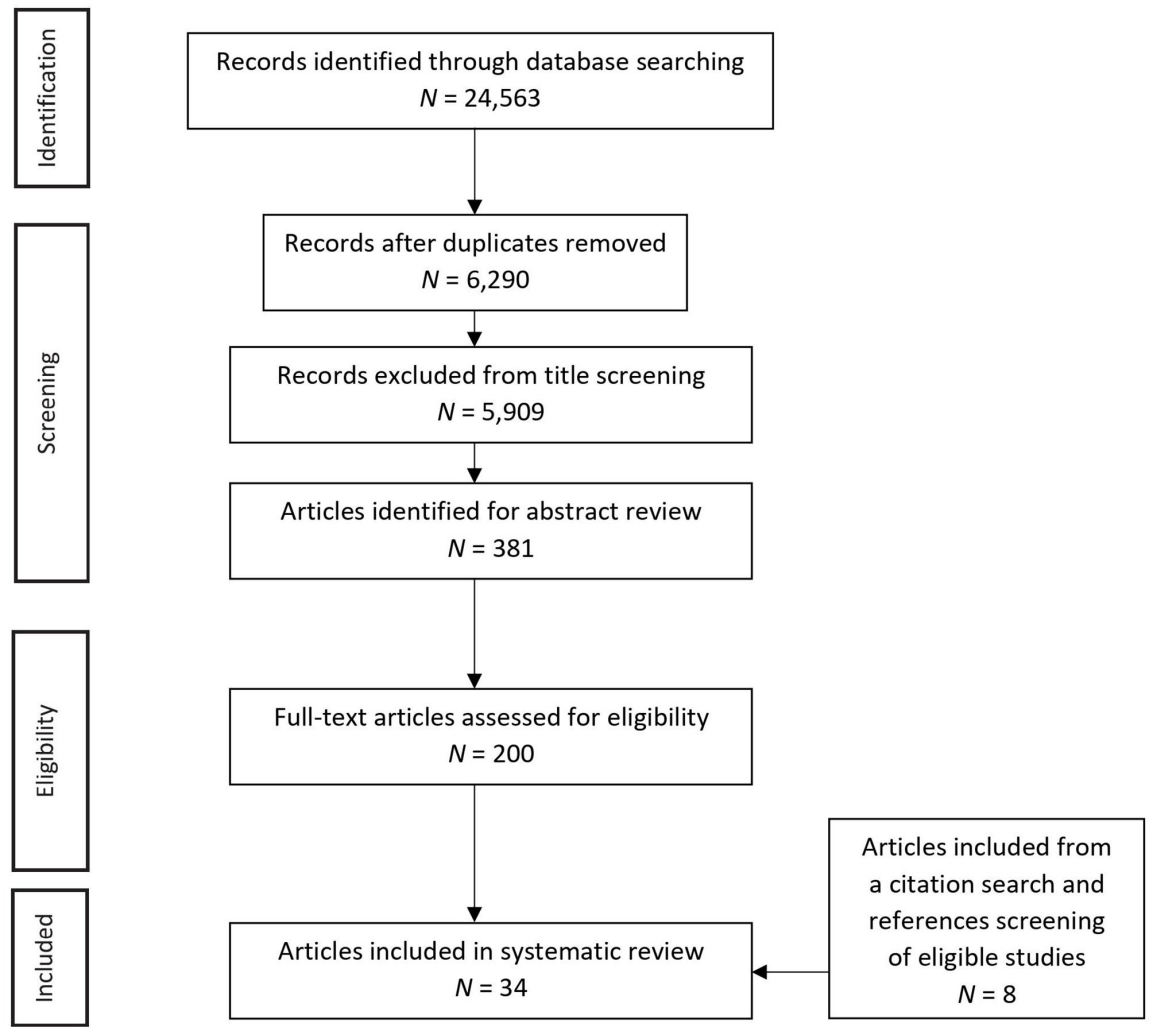
Paper selection flow chart.

The literature search yielded 24,563 articles. After electronically removing duplicates, the 6,290 remaining studies were screened based on title and abstract, and 6,090 articles were excluded as ineligible. The remaining 200 articles were assessed at full-text level, and 174 were excluded. An additional eight articles were included from a citation search and screening of the references of eligible studies. This resulted in 34 articles being included in the systematic review (see Figure 1). Table 1 provides an overview of the studies reviewed, including details of the research measures employed. Papers were from the USA (*n* = 18), Canada (*n* = 2), Spain (*n* = 2), Germany (*n* = 2), the Netherlands (*n* = 2), Belgium (*n* = 2), Iran (*n* = 2), the UK (*n* = 1), Norway (*n* = 1), Japan (*n* = 1), and Turkey (*n* = 1). Cross-sectional (*n* = 24), longitudinal (*n* = 9), and case–control (*n* = 1) designs were included.

The age range of adult samples varied substantially across eligible studies from 18 to over 65 years, young adults from 16 to 29 years, older adults from 62 to 90 years, and adolescent samples from 11 to 19 years. Thus, there was no single definition of adolescent, young adult, older adult, and adult samples in this review, and these descriptions were instead based on how the original studies defined their samples. Overall, eligible articles comprised 14,332 adults, 4,116 young adults, 81 older adults, and 3,434 adolescents, with a total of 888 clinical participants involved. Eight studies used adolescent samples, 26 studies used adult samples, nearly half of which were young adults recruited from universities (*n* = 11), and only one (51) investigated older adults. Among all eligible studies, only seven used clinical samples (28, 63, 69, 74, 75, 80, 85). Most of the studies included both men and women; four studies (21, 32, 64, 71) included female participants only and one study (52) included male participants only.

Sample sizes ranged from 50 to 5,614 participants. Twenty-nine studies had sample sizes larger than 100. We used a sample size of 100 as guideline for performing mediation analyses, as MacKinnon et al. (90) argued that a sample size of 100–200 was sufficient even for multiple mediator models in terms of power. Nine studies examined more than one mediating variable in their analyses (36, 79). Of the 34 studies that tested mediation, seven were rated as weak, three as moderate, and 24 as strong. More than one-third (*n* = 13) studies lacked information or did not provide an interpretation for potential confounding variables, such as age, gender, and socioeconomic status. Eleven studies controlled for other types of childhood maltreatment (e.g., sexual and physical abuse) in the mediation analyses to disentangle the independent contribution of emotional abuse.

Among all eligible studies, six (17.6%) found no association between childhood emotional abuse, mediating variables, and subsequent depression levels in their analyses. Van Assche et al. (51) reported that childhood emotional abuse was not significantly correlated with current depression or attachment in a sample of community-dwelling older adults. Carvalho Fernando et al. (63) found no significant impact of self-reported childhood emotional abuse and emotion dysregulation on depressive symptoms. Hayashi et al. (69) reported that negative life events were affected by childhood abuse but did not predict the severity of depression. Kaysen et al. (71) showed no association of childhood emotional abuse with either adult depression or maladaptive cognitions. Calvete (62) reported that parental emotional abuse did not predict the worsening of early maladaptive schemas in adolescence, although it was directly associated with depressive symptoms. Jessar et al. (70) also indicated that emotional abuse did not significantly predict deficits in emotional clarity but did predict increases in depressive symptoms.

In addition, no specificity was found in the relation between emotional abuse and anhedonic depression in Lumley and Harkness's (28) analysis. Unexpectedly, Schulz et al. (80) reported that childhood emotional abuse was not correlated with expert ratings of depression, although it was correlated with self-rated depression severity. Some of these findings might be caused by the considerable co-occurrence of childhood sexual, physical, and emotional abuse in the studied samples. Any independent effects of emotional abuse on the development of depression might be diminished when severe physical or sexual abuse is also present. Therefore, we suggest that it would be important for future research to assess exposure to all forms of childhood maltreatment simultaneously, as multiple types of maltreatment may interact to produce varying outcomes, and to rule out other forms of maltreatment that may have been contributing factors when examining the emotional abuse–depression link.

### Overview of the Measures Used in the Primary Studies

#### Childhood Emotional Abuse

Following methodological recommendations from PRISMA (49), a component approach to quality assessment was employed. Regarding the measures used in the eligible studies, reliability and validity were taken into consideration. According to the EPHPP, (a) standard assessment tools with known reliability and validity were rated as strong; (b) data collection tools that have been shown to be valid but not shown to be reliable (or for which reliability is not described) were rated as moderate; and (c) measures that have not been shown to be valid (or for which both reliability and validity are not described) were rated as weak. Four studies obtained weak ratings for measures employed to capture childhood emotional abuse. The remaining studies employed valid and reliable measures of the independent variables being investigated. The most widely used retrospective measures of childhood emotional abuse were the CTQ (91, 92) (*n* = 14) and the LEQ (93) (*n* = 6). Research has provided considerable support for the reliability and validity of both scales. The CTQ is a 28-item self-report inventory developed to measure five types of abuse or neglect in childhood or adolescence. Five CTQ items directly assess verbal and non-verbal emotional abuse: (1) People in my family called me things like “stupid,” “lazy,” or “ugly”; (2) I thought that my parents wished I had never been born; (3) People in my family said hurtful or insulting things to me; (4) I felt that someone in my family hated me; (5) I believe that I was emotionally abused. Participants rate each item on a 5-point Likert-type scale with higher scores indicating more emotional abuse. The CTQ also provides cut-off scores for none to low, low to moderate, moderate to severe, and severe to extreme trauma exposure, and the cut-off scores for moderate to severe maltreatment are 13 or higher for emotional abuse (92). The LEQ scenarios describe specific examples of emotional, physical, and sexual abuse. Participants are instructed to indicate whether these events had occurred, the age of onset for each maltreatment event, cessation of the event, the frequency of occurrence, and the perpetrator, rather than reporting on global estimates of abuse. Twenty-seven items in the LEQ assess emotional abuse, including forms of belittling, ridiculing, spurning, humiliating, rejecting, extorting, and terrorizing. For example, “Did anyone humiliate or demean you in the presence of other people?” Only three studies adopted interview measures. Two studies (28, 31) used the CECA (94). The CECA is a semi-structured contextual threat interview that assesses childhood adversity, including the familial context of quality of care and relationship with parents or substitutes (i.e., antipathy, emotional neglect, discipline, discord) in addition to psychological, physical, and sexual abuse.

#### Depression

The operationalization of depression varied greatly. Using EPHPP criteria, one study (77) obtained a weak rating for the measure employed to capture depression symptoms; the remaining studies employed valid and reliable measures of the dependent variables being investigated. Most studies examined symptoms of depression as the outcome variable in community samples. Among the 34 eligible studies, only seven were conducted with clinical samples (28, 63, 69, 74, 75, 80, 85). Seventeen studies used the BDI and the BDI-II (95, 96), four used the CES-D (97), two used the DASS-21 (98), two used the MASQ (99), and two used the CDI (100).

Nine studies (21, 28, 66, 71, 74, 79, 81, 83, 89) used interview measures to assess the severity of depression or diagnosis. Four studies (21, 71, 74, 89) were conducted with different versions of the SCID (101) to assess major depressive disorder (MDD) as defined in DSM-IV (102). Three studies (74, 79, 83) used the CIDI (103) also based on DSM-IV criteria for MDD (102). Three studies (28, 66, 81) used different versions of the SADS (104) to assess episodes of depression.

#### Mediators

A range of potential mediators was assessed by various measures. According to the EPHPP, five studies obtained weak or moderate ratings for measures employed to capture the mediating variable; the remaining studies employed valid and reliable measures of the mediators being investigated.

### Synthesis of the Mediators of the Emotional Abuse–Depression Relationship

**Figure 2:**
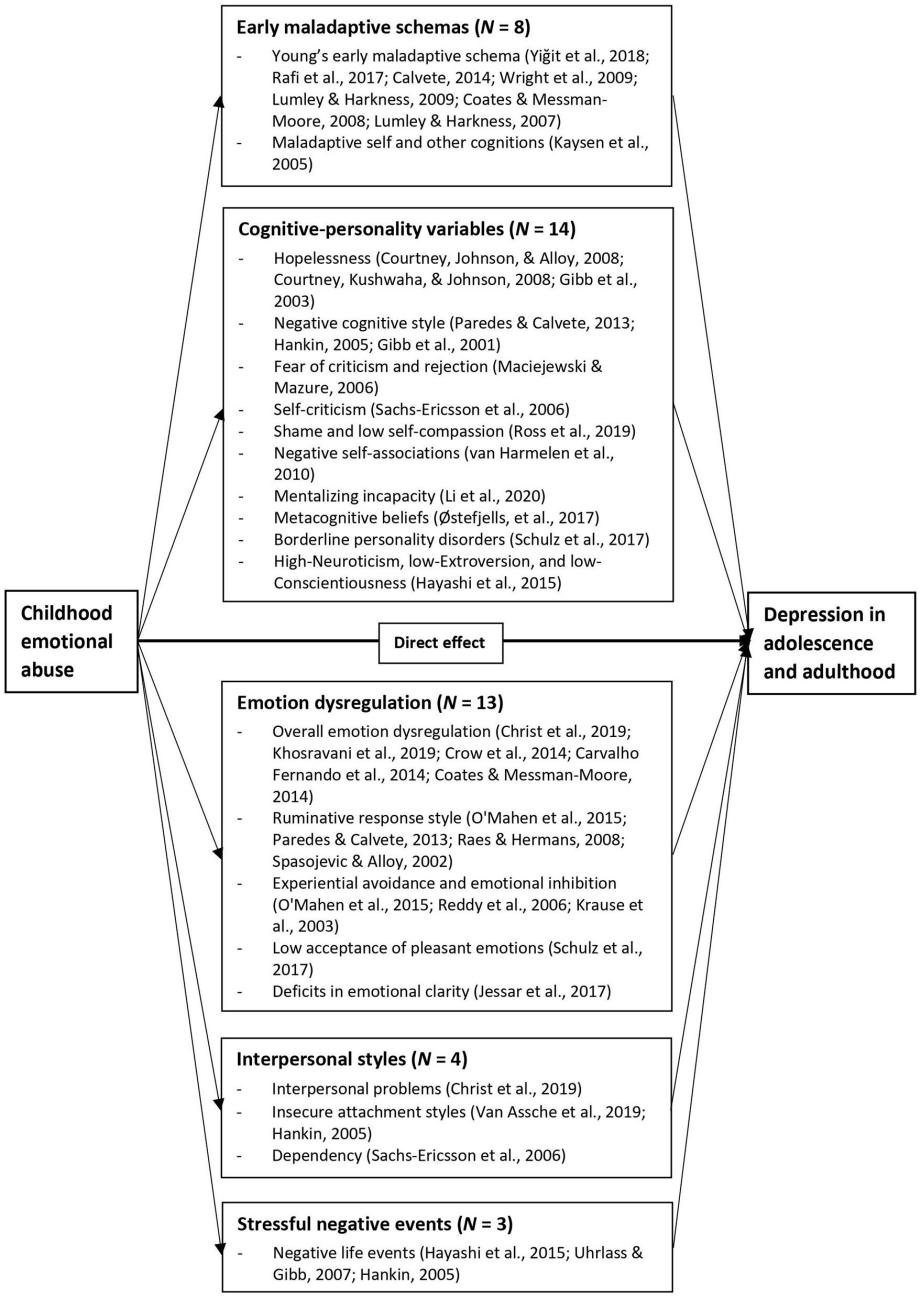
Examined mediators in the emotional abuse–depression link.

For the purposes of the review, we grouped studies according to the type of psychological mechanisms examined in the emotional abuse–depression link. These mediators were further grouped according to the five clusters of psychological mediators: early maladaptive schemas, cognitive-personality variables, emotion dysregulation, interpersonal styles, and stressful negative events (see Figure 2). These domains were created as part of the narrative integration approach we used to organize the findings from the literature. Eight studies had analytic approaches rated as weak (e.g., regression methods) on their appropriateness in terms of testing for mediation effects, three studies were rated as moderate (e.g., regression methods with additional Sobel test), and the remaining 23 studies were rated as strong (e.g., bootstrapping techniques; path analysis). Table 3 provides a summary of results and mediational analytic approaches.

**Table 3:** Summary of results and mediational analytic approaches.

**Cluster**	**Eligible studies**	**Percentage of studies supporting the pathway**	**Strength of the mediation analysis**		
			**Strong**	**Moderate**	**Weak**
Early maladaptive schemas (*N* = 8)	Calvete (62) Coates and Messman-Moore (64) Kaysen et al. (71) Lumley and Harkness (28) Lumley and Harkness (89) Rafi et al. (77) Wright et al. (84) Yigit et al. (85)	75%	*N* = 5	*N* = 1	*N* = 2
Cognitive-personality variables (*N* = 14)	Courtney et al. (65) Courtney et al. (30) Gibb et al. (66) Gibb et al. (67) Hankin (31) Hayashi et al. (69) Li et al. (54) Maciejewski and Mazure (74) Østefjells et al. (75) Paredes and Calvete (36) Ross et al. (53) Sachs-Ericsson et al. (79) Schulz et al. (80) van Harmelen et al. (83)	92% (*N* = 12; cognitive vulnerability—the small bandwidth of cognitive-personality variables) 100% (*N* = 2; personality—the broader cognitive-personality variables)	*N* = 9	*N* = 1	*N* = 4
Emotion dysregulation (*N* = 13)	Carvalho Fernando et al. (63) Christ et al. (32) Coates and Messman-Moore (64) Crow et al. (29) Jessar et al. (70) Khosravani et al. (52) Krause et al. (72) O'Mahen et al. (21) Paredes and Calvete (36) Raes and Hermans (76) Reddy et al. (78) Schulz et al. (80) Spasojevic and Alloy (81)	85%	*N* = 10	*N* = 1	*N* = 2
Interpersonal styles (*N* = 4)	Christ et al. (32) Hankin (31) Sachs-Ericsson et al. (79) Van Assche et al. (51)	50%	*N* = 3	*N* = 1	N/A
Stressful negative events (*N* = 3)	Hankin (31) Hayashi et al. (69) Uhrlass and Gibb (82)	67%	*N* = 3	N/A	N/A

*Nine studies examined more than one mediating variable in their analyses so might be counted more than once in different clusters in this table*.

First, a large body of cross-sectional and prospective research has highlighted the role of negative schema contents in depression. Early maladaptive schemas (105) are defined as broad, dysfunctional, and pervasive patterns consisting of memories, emotions, cognitions, and bodily sensations about oneself and relationships with others and hypothesized to consolidate over time. Certain schema domains (disconnection/rejection, impaired autonomy and performance, and other directedness) have been found to be associated with depressive symptoms (106).

Second, theories that focus on personality dimensions include broad-bandwidth theories focusing on the Big Five dimensions (107), and narrower bandwidth theories focusing on more specific personality dimensions, such as sociotropy–autonomy (108, 109) and cognitive vulnerability as conceptualized in Beck's original formulation of the cognitive model of depression (108, 110). In this review, we grouped cognitive vulnerability with personality traits and dysfunctions as cognitive-personality variables. Cognitive theories of depression (111) have postulated that the impact of childhood maltreatment on subsequent psychopathology might be mediated by cognitive vulnerability (i.e., relatively stable, trait-like negative cognitive styles). People with chronic experiences of childhood emotional abuse, as opposed to physical or sexual abuse, are more likely to interpret current negative events in a similarly depressogenic manner (e.g., negative self-associations) because the depressogenic interpretations are directly supplied by the abusers (e.g., “you are such a stupid child”), which accounts for subsequent increases in depressive symptoms (61). A large number of studies have shown dysfunctional attributional styles and negative inferential styles for causes, consequences, and self-characteristics as predictors of depression in clinical and non-clinical samples (112–114). In addition, high Neuroticism, low Extroversion, and low Conscientiousness in the five-factor model of personality have been consistently linked to the onset of MDD (115). Although traits are considered to be largely genetically determined, neuroticism has been found to change over time as a result of childhood maltreatment (116). Several studies also revealed associations between childhood emotional abuse and different areas of personality dysfunction, including borderline, avoidant, and dependent pathology (117, 118). Given the high prevalence of comorbid personality disorders in inpatients with MDD (119), changes in personality functioning may mediate the emotional abuse–depression link. In Beck's (108) models of depression, these cognitive-personality characteristics have been shown not to be simply latent diatheses for depression; instead, they increase the probability of particular life events and one's vulnerability for the onset of depression with a diathesis-stress nature (120).

Third, chronic experiences of parental emotional abuse may increase children's internal focus and lead to emotion dysregulation, such as rumination. Individuals who tend to ruminate in response to negative life events are at high risk of developing and maintaining depressive symptoms. Treynor et al. (121) identified two distinct rumination components, labeled as brooding and reflection. There is a body of research consistently linking brooding rumination, but not reflection, with the onset and maintenance of depression (122).

Fourth, by definition, emotional abuse encompasses a repeated pattern of maladaptive interactions between the parent and child and has been described as a “pathogenic relational environment” [(123), p. 414]. Exposure to emotional abuse clearly threatens the security of attachment relationships and results in maladaptive models of self and self-in-relation-to-others, which may then impact on the quality of later interpersonal relationships. Individuals with a history of childhood maltreatment in general reported more dysfunctional relationships (124) and lower levels of social support (34) in adulthood. Recurring frustrated interpersonal dynamics, in turn, will render an individual more vulnerable to depression (125). Although empirical research is still scarce, interpersonal problems have been thought to be an underlying factor of the emotional abuse–depression link (126, 127).

Finally, Hammen's (128, 129) stress generation model of depression posited that a reciprocal relationship exists between negative life events and depression, whereby depressed individuals may generate additional negative stressors as they seek reassurance and comfort from others, only to be rejected by those they turned to for social support, and thus actively contribute to the occurrence of negative events in their lives. Importantly, Hammen's model suggests that childhood emotional abuse may set the stage for self-generated negative events and depressive symptoms in adolescence and adulthood (130).

#### Early Maladaptive Schemas

In total, eight studies examined early maladaptive schemas as the pathway by which childhood emotional abuse may lead to subsequent depression symptoms. Both cross-sectional (28, 64, 71, 77, 84, 85, 89) and longitudinal (62) designs were employed. Five (28, 62, 64, 84, 89) used the YSQ (105) and two (77, 85) adapted the YSQ for Turkish or Iranian adolescents.

Four studies examined particular schema themes that may mediate the emotional abuse–depression link (28, 77, 84, 85). Lumley and Harkness (28) found that specific early maladaptive schemas of social isolation and self-sacrifice mediated the relation between early emotional maltreatment and anhedonic depression in depressed adolescents. Wright et al. (84) found that the schemas of vulnerability to harm, self-sacrifice, and defectiveness/shame mediated the relationship between childhood emotional abuse and symptoms of depression in a general population of young adults. In Turkish adolescents, Yigit et al. (85) found that both disconnection/rejection and impaired autonomy significantly mediated emotional abuse and depression in their non-clinical sample, whereas only disconnection/rejection significantly mediated the relationship between emotional abuse and depression in their clinical sample. In Iranian adolescents, Rafi et al. (77) reported that early maladaptive schemas of loneliness, vulnerability to harm, and submission mediated the relationship between childhood emotional maltreatment and depression. However, neither the Child Abuse Self Report Scale (CASRS) for assessing childhood emotional maltreatment nor the Schema Inventory for Children (SIC) for measuring early maladaptive schemas used in the study of Rafi et al. was validated. The CASRS was created in a master's thesis (131) and not peer-reviewed or published, and no further evidence of validity or reliability was found for the SIC.

In another two studies using the YSQ, Coates and Messman-Moore (64) found that general negative internalized beliefs significantly mediated the link between childhood psychological maltreatment and depressive symptoms. Lumley and Harkness (89) used a computer task, the Modified-Psychological Distance Scaling Task (M-PDST), whose stimuli (i.e., schema statements) were derived from the YSQ, to examine depressotypic schema organization as a mediator in young adults. They found that tightly connected negative schema statements mediated the relation between maternal and paternal emotional maltreatment and current depression, whereas loosely connected positive schema statements mediated the relation between maternal emotional maltreatment and current depression.

Unexpectedly, two studies (62, 71) found no association of parental emotional abuse with depression and/or maladaptive schemas. Calvete (62) reported that parental emotional abuse predicted adolescents' depressive symptoms but not a worsening of early maladaptive schemas over time. Regardless, parental emotional abuse might have influenced the origin of early maladaptive schemas before their study. Kaysen et al. (71) found no association of childhood emotional abuse with depression or maladaptive cognitions in a sample of adult women who had recently been raped or physically assaulted. However, they used only three items (i.e., being called bad, dumb, or stupid; being threatened with a beating; being cursed at) of the HVQ to measure experiences of childhood emotional abuse in a recently traumatized population, and did not rule out other forms of maltreatment that are very likely to be contributing factors. This limits the individual effects of childhood emotional abuse on subsequent maladaptive cognitions and depressive symptoms.

Overall, six out of eight (75%) studies (28, 64, 77, 84, 85, 89) supported early maladaptive schemas as the pathway by which childhood emotional abuse may lead to subsequent depression symptoms. However, there is a lack of consistency in their findings concerning whether maladaptive schemas in general or specific schema themes act as the mediator. Only the schemas of vulnerability to harm and self-sacrifice appeared as the mediators in the emotional abuse–depression link more than once. Overall, two studies were rated as weak (71, 84), one as moderate (89), and five as strong, in terms of the relative strength of the statistical mediation analysis.

#### Cognitive-Personality Variables

In total, 14 studies examined cognitive-personality variables as the pathway by which childhood emotional abuse may lead to subsequent depression symptoms. Within this domain, we split cognitive-personality variables into two subgroups (narrow bandwidth vs. broad bandwidth): one collection of studies (*n* = 12) has drawn on cognitive processes focusing on specific types of cognitive vulnerability, whereas another tradition has focused on broader cognitive-personality factors (*n* = 2).

In the subgroup of studies focusing on cognitive vulnerability, three studies (30, 65, 67) reported hopelessness as a mediator in the emotional abuse–depression link in adolescent or young adult samples using the BHS. Courtney et al. (65) found that the association of emotional abuse with depressive symptoms remained significant even after controlling for hopelessness, indicating that hopelessness was a partial mediator of the association between emotional abuse and depressive symptoms. In line with them, Gibb et al. (67) also indicated that the etiological chain of the hopelessness theory mediates only part of the relation between emotional maltreatment and symptoms of hopelessness depression. However, Courtney et al. (30) showed that hopelessness accounted for 87.3% of the variance in this association where the association of emotional abuse index scores with depression did not remain significant after controlling for hopelessness. Nonetheless, Courtney et al. (30) used only three items adapted from the CTQ that directly assess verbal and non-verbal emotional abuse and did not rule out other forms of maltreatment that may have been contributing factors, which limits the individual effects of emotional abuse on subsequent hopelessness and depressive symptoms.

Three studies (31, 36, 66) examined cognitive vulnerability as a potential mediating variable using the CSQ. Gibb et al. (66) found that cognitive risk fully mediated the relation of childhood emotional abuse to non-endogenous major depression and hopelessness depression, respectively, in a 2.5-years follow-up study. Hankin (31) also found that cognitive vulnerability helped account for the emotional abuse–depression link in two separate studies, a 10-weeks and a 2-years longitudinal study with undergraduate students. However, Paredes and Calvete (36) showed no evidence for negative inferential styles to be a mediator in their follow-up study. They reported that only negative inferential styles for consequences predicted depressive symptoms, which notably contrasts with studies showing that inferential style for causes of negative events is the most important element in the prediction of depressive symptoms (112, 113, 132). The CSQ employed in the above three studies is a self-report instrument assessing explicit cognitions. To include both explicit and automatic processes of cognitive functioning, van Harmelen et al. (83) had 2,837 adult participants complete a computer task, the Implicit Association Test (IAT). They found that childhood emotional maltreatment had the strongest link with enhanced explicit and automatic self-depression associations, compared with physical and sexual abuse; moreover, an increase in explicit and automatic negative self-associations partially mediated the relation between childhood emotional maltreatment and depressive symptomatology.

Three studies (53, 74, 79) assessed specific negative cognitive styles as mediators in the emotional abuse–depression link: fear of criticism and rejection (74), self-criticism (79), and shame and low self-compassion (53). Self-criticism, self-blame, and non-compassionate introjects may underlie the feelings of shame with a harsh internalized voice. All of them were found to significantly mediate the emotional abuse–depression link in adult samples. A significant path from emotional abuse to depression and a significant indirect path that passed through self-compassion and shame were reported by Ross et al. (53). However, after adjusting for fear of criticism and rejection, perceived childhood emotional abuse was no longer significantly associated with major depression in another study (74). Although Sachs-Ericsson et al. (79) also reported that verbal abuse no longer predicted depression once self-criticism was included in the analyses, they used specific behaviors of verbal abuse (e.g., insulted, swore at, did or said something to spite, threatened to hit) to estimate emotional abuse, which was neither validated nor shown to be reliable.

Finally, two studies (54, 75) examined metacognition as a mediator in the emotional abuse–depression link. Li et al. (54) found that childhood emotional abuse continued to exert a significant effect on adulthood depression after controlling for other forms of early maltreatment and current mentalizing incapacity in a general sample of 205 adults. A mediation effect between childhood emotional abuse and adulthood depression symptoms via current mentalizing incapacity (both hypermentalizing and hypomentalizing) was established in their analysis. Østefjells et al. (75) reported that specific beliefs about thoughts being uncontrollable and dangerous significantly mediated the relationship between early emotional abuse and depression in 261 adult patients with psychotic or bipolar disorders. Although both studies examined metacognition-related mediators, Li et al. (54) introduced mentalizing to capture the interpersonal, cognitive, and developmental constructs of depression as an umbrella term for a group of basic psychological processes (e.g., theory of mind, reflective functioning), whereas metacognitive beliefs in Østefjells et al. (75) cover only specific beliefs about thoughts being uncontrollable and dangerous.

In the subgroup with broad bandwidth, two studies examined personality as a mediator in clinical adult samples by employing a cross-sectional (69) and a prospective observational (80) design. Hayashi et al. (69) found that childhood emotional abuse predicted the severity of depression indirectly through the mediation of high Neuroticism, low Extroversion, and low Conscientiousness in Japanese MDD patients. Schulz et al. (80) reported that elevated borderline personality traits mediated the association of childhood emotional abuse and self-rated MDD symptoms in German MDD patients; additionally, elevated passive-aggressive personality disorder traits mediated the link between childhood emotional abuse and lower self-rated symptom improvement. Although both studies examined personality as a potential mediator, Hayashi et al. (69) measured the five personality domains described in the five-factor model (107) using the NEO-FFI, whereas Schulz et al. (80) assessed personality traits using the PSDI. Unlike the NEO-FFI, the PSDI allows assessment of DSM-IV and ICD-10 personality traits as a dimensional approach in which characteristics of specific personality traits vary from adaptive to clinical levels and further reflect a personality disorder. Hayashi et al. (69) conducted structural equation modeling to examine the mediation model, and Schulz et al. (80) estimated direct and indirect effects using Preacher and Hayes (73) and the Sobel test; both were therefore rated as strong.

Overall, 11 of 12 (92%) studies drawing on cognitive processes established cognitive vulnerability, including hopelessness, general and specific types of negative cognitive styles, and metacognition, as mediators in the emotional abuse–depression link. Two studies assessing personality as a mediator potentially supported broader cognitive-personality factors as the pathway. There appears to be consistency of evidence supporting cognitive vulnerability (the small bandwidth of cognitive-personality variables; *n* = 11) as the pathway based on a noticeably greater number of studies, in contrast to personality (the broader cognitive-personality variables; *n* = 2). Although the above outcomes suggest that cognitive vulnerability may represent a common mediating mechanism in the emotional abuse–depression link, four were rated as weak (30, 65, 66, 74) and one as moderate (79) in terms of the analytic approach employed to test for mediation effects. Hence, further research is needed to replicate the abovementioned outcomes with explicit analyses estimating direct and indirect effects (e.g., bootstrapping techniques).

#### Emotion Dysregulation

In total, 13 studies examined emotion dysregulation as a potential mediating variable in the emotional abuse–depression link. Overall emotion dysregulation was examined in five studies (29, 32, 52, 63, 64). Two studies (32, 64) reported that the effect of childhood emotional abuse on depressive symptoms was significantly mediated by emotion dysregulation measured by the DERS in emerging adult women. Khosravani et al. (52) reported a direct effect of emotional abuse on depressive symptoms and an indirect effect via emotion dysregulation, also measured by the DERS, in a sample of 350 treatment-seeking males with heroin dependence. Consistently, Crow et al. (29) found that emotion dysregulation partially mediated the relationship between childhood emotional abuse and later depression in a low-income African American sample of 3,902 adults, using the EDS. However, no significant impact of self-reported childhood emotional abuse and emotion dysregulation on depressive symptoms was found in a case–control sample by Carvalho Fernando et al. (63), although they reported that a history of emotional abuse was uniquely related to more frequent use of expressive suppression.

Eight studies examined specific dysfunctional emotion regulation strategies (e.g., rumination, emotional inhibition, experiential avoidance, low acceptance of pleasant emotions) as potential mediating variables. These dysfunctional emotional regulation strategies are largely overlapping and mutually influencing constructs. Reddy et al. (78) found that increased levels of experiential avoidance significantly mediated the relationship between childhood psychological abuse and current symptoms of depression in a cross-sectional sample of 987 undergraduates, reducing the direct effect by 77%. Experiential avoidance contains the elements of emotional inhibition and behavioral avoidance. In support of Reddy et al. (78), Krause et al. (72) found that chronic emotional inhibition fully mediated the relationship between a history of parental psychological abuse and adult depressive symptoms in a cross-sectional study. While employing an experientially avoidant coping style to inhibit thoughts and feelings may be temporarily adaptive as a means of escaping aversive emotional experiences, it often paradoxically leads to increased rumination on the thing sought to be avoided (78).

A ruminative response style was specifically examined as a potential mediating variable in four studies (21, 36, 76, 81). Spasojević and Alloy (81) first examined and found that rumination partially mediated the relationship between childhood emotional maltreatment and major depressive episodes in young adults who were followed prospectively for 2.5 years. The other three studies (21, 36, 76) all reported that brooding rumination (not reflection) partially mediated the relationship between emotional abuse and depressive symptoms in samples of adolescents, young adults, and low-income pregnant women. They suggested that the mediating role of rumination would be attributable to the brooding form, rather than to the more adaptive component of reflection. As the relationship between brooding and behavioral avoidance was hypothesized to be reciprocal (133), O'Mahen et al. (21) also examined behavioral avoidance in their study but found no correlation between childhood emotional abuse and behavioral avoidance.

Moreover, Schulz et al. (80) reported that low acceptance of pleasant emotions mediated the association between childhood emotional abuse and self-rated depressive symptoms in a longitudinal study of 123 German MDD inpatients. Finally, Jessar et al. (70) assessed emotional clarity as a mediator between childhood emotional maltreatment and adolescent depression in a longitudinal study, but found that emotional abuse did not significantly predict deficits in emotional clarity.

In total, 11 of 13 (85%) studies in this cluster supported emotion dysregulation as a mediator in the emotional abuse–depression link. Overall emotion dysregulation (*n* = 4) and brooding rumination (*n* = 4) appeared to be the most consistent mediators in this link. In terms of the analytic approach employed to test for mediation effects, all studies in this cluster scored strong, except for two studies (63, 81) rated as weak, and one study (76) rated as moderate.

#### Interpersonal Styles

Four studies (31, 32, 51, 79) examined interpersonal styles as the pathway by which childhood emotional abuse may lead to subsequent depression symptoms. Hankin (31) reported that a more insecure attachment style prospectively (over both 10-weeks and 2-years follow-up) mediated the association between a childhood history of emotional maltreatment and depressive symptoms in young adults. However, in a sample of older adults aged 62 and older, Van Assche et al. (51) found no significant correlation of childhood emotional abuse with either insecure attachment or current level of depression. Christ et al. (32) found that the effect of childhood emotional abuse on depressive symptoms was significantly mediated by two domains of interpersonal problems: cold/distant and domineering/controlling. Finally, Sachs-Ericsson et al. (79) unexpectedly found that dependent characteristics did not mediate the relationship between any parental abuse and depression symptoms in a large sample of adults, although a dependent interpersonal style has been constantly posited as a vulnerability marker for depression in literature. Three studies scored strong in their mediational analytic approaches (31, 32, 51) and one (79) was rated as moderate. Overall, only half of the studies in this cluster (31, 32) found expected outcomes supporting insecure attachment styles and specific interpersonal styles as potential pathways in the emotional abuse–depression link.

#### Stressful Negative Events

Three studies examined stressful negative events as a potential mediating variable in the emotional abuse–depression link (31, 69, 82). Two studies (31, 82) employed a longitudinal design with path analysis in young adults. Uhrlass and Gibb (82) found that changes in recent negative events fully mediated, rather than moderated, the link between childhood emotional maltreatment and depressive symptoms; additionally, initial depressive symptoms contributed to prospective changes in negative life events. Instead of assessing major life events (e.g., deaths or unemployment), the Hassles Scale used in Uhrlass and Gibb's 7-weeks longitudinal study calculated the level of negative life events by summing the number of hassles endorsed during the past week. Hankin (31), described above, also assessed negative life events as a potential mediator using NLEQ which examines negative life events that typically occur for young adults. They found that experiencing a greater number of negative life events prospectively (over both 10-weeks and 2-years follow-up) mediated the association between a childhood history of emotional maltreatment and depressive symptoms in young adulthood. Unexpectedly, Hayashi et al. (69) found that negative life change affected by childhood emotional abuse did not predict the severity of depression. Although these three studies all scored strong in their mediational analytic approaches, only two of them supported stressful negative events as a potential mediating variable.

## Discussion

Existing reviews (24, 33, 39, 40) have focused on the psychological mechanisms leading from childhood maltreatment in general to adult depressive symptoms, without considering the specific contributions of subtypes of maltreatment. The current systematic review goes beyond previous findings by examining potential mediating variables specifically in the emotional abuse–depression link. The aims of this review were to provide a comprehensive systematic review of quantitative literature investigating potential psychological mediators of the association between childhood emotional abuse and dimensional and categorical depression symptoms, with a focus on the quality of this evidence, including the relative strength of the statistical mediation analyses used to explain the emotional abuse–depression link. Our findings demonstrate several potentially causal mechanisms that might be involved in the relation between childhood emotional abuse and adolescent and adult depression. These psychological mediators fall into five clusters: (1) early maladaptive schemas, (2) cognitive-personality variables, (3) emotion dysregulation, (4) interpersonal styles, and (5) stressful negative events.

An important limitation of the eligible studies lies in the fact that not all of them addressed the unique impact of emotional abuse by accounting for the effects of the other types of childhood maltreatment. Only one-third of studies (*n* = 11) identified the unique contribution of emotional abuse to depression by statistically controlling for a history of other forms of childhood maltreatment (29, 31, 32, 53, 54, 63, 78–80, 83, 84). Emotional abuse was found to be uniquely associated with later depression after partialing out the effect of other forms of childhood maltreatment, and the findings of these 11 studies remained essentially unchanged when covariates were removed. Wright et al. (84) reported that the intercorrelations between all the abuse variables ranged from a low of 0.16 (between sexual abuse and emotional neglect) to a high of 0.63 (between physical abuse and emotional abuse), suggesting that different forms of child maltreatment do covary with each other and often co-occur in the same household. Given the co-occurring forms of childhood abuse, neglect, and other adverse family experiences, it would be of importance for future mediational studies to model the concurrent and possible additive effects of different types of abuse. In addition, a few studies (72) did not report the strength of the direct effect between the emotional abuse latent variable and the depression latent variable without the mediator latent variable in the model. As such, a level of caution is required in interpreting the relative contributions of emotional abuse and mediators to ongoing depression.

Most eligible studies focused on examining a single mediator and indicated that this particular factor had the most impact in leading to depression following childhood emotional abuse. Nine studies included more than one variable in the mediational model of the emotional abuse-depression link (21, 31, 32, 36, 53, 64, 69, 79, 80), mainly involving emotion dysregulation, negative cognitive styles, stressful negative events, interpersonal styles, and personality, suggesting that the pathway from emotional abuse to depression is multi-determined and involves both cognitive and emotion-related factors. However, when integrating more than one mediator in a predictive model, not only effects and causal inferences might overlap with each other, but bidirectional relationships might also exist among contributing variables. For example, studies on stress generation (106, 134) have suggested that temperament may be related to stress-generation and increase interpersonal difficulties. Moreover, the cognitive and emotional deficits in the development of depression are not necessarily exclusive; studies have suggested an important role of cognitive processes underlying the regulation of emotion (135). Coates and Messman-Moore (64) reported that negative internalized beliefs increased vulnerability to emotion dysregulation and showed that the latter was an eventual outcome in mediating the emotional abuse-depression link.

Furthermore, as some mediating variables may be significant only when tested in isolation and not when entered simultaneously with other mediating variables, controlling for the interrelation between psychological mediators is important. Hankin (31), for instance, reported that an insecure attachment style and negative life events almost completely mediated the association between childhood emotional abuse and later depressive symptoms, while a negative cognitive style was minimized in their multivariate mediational model. Similarly, Coates and Messman-Moore (64) found that negative internalized beliefs no longer predicted depressive symptoms where emotion dysregulation appeared to be the stronger factor when the two were simultaneously entered into the same model. Likewise, a ruminative response style consistently appeared to be a stronger factor when entered simultaneously with cognitive vulnerability competing in the same mediational model (36, 81). Thus, our review supports the notion that not one mediator in isolation, but complex interrelations of psychological mediators, affect the relationship between childhood emotional abuse and subsequent depression.

Finally, although this review has shown specific pathways from exposure to childhood emotional abuse to later depression, the effects of childhood adversities are considered multivariate in their ultimate presentation (136). It has been suggested that childhood emotional abuse could first lead to the onset of depression in children and adolescents, and that depression in childhood and adolescence confer risk for depression in adulthood (137). A distal history of childhood maltreatment and context does not invariably contribute to depressive symptoms in adolescence and adulthood. Hamilton et al. (138) found that emotional abuse occurring during adolescence contributed to depressive symptoms in this period. Liu et al. (15) found that ongoing emotional maltreatment predicted shorter time to first onset of MDD in a sample of young adults. These findings together suggest that although childhood emotional abuse may have a specific depressogenic effect over a longer period of time, emotional abuse that occurs during that developmental stage may contribute to depression in a more immediate period. Further research is required to determine the specific contextual and process-related factors underlying this relationship.

### Toward a More Comprehensive Theoretical Model

In most eligible studies reporting mediations, childhood emotional abuse still significantly predicted depression symptoms after accounting for potential mediators, suggesting that each examined mediator is only one of many potential pathways in the emotional abuse–depression link. However, the psychological mediators examined in the emotional abuse–depression link in previous studies are largely dependent on researchers' preferences. Moreover, there are big differences in the conceptual and epistemological status of the various mediators that have been investigated. Person-dependent stressful life events, for instance, focus on the interplay between the individual and his/her environment, while rumination focuses on intrapsychic processes. Yet, it must be clear that rumination may also contribute to stress-generation processes, as do higher order theoretical constructs, such as personality. Hence, the current review suggests that there is a great degree of overlap in the investigated mediators between childhood emotional abuse and depression in adolescence and adulthood. Given that current theories on the pathways from childhood emotional abuse to subsequent depression (e.g., the hopelessness theory of depression and the stress generation theory of depression) are based on isolated investigations of researchers' interests rather than systematic empirical studies, this systematic review may contribute to a move to empirical research based on a more comprehensive theoretical model that incorporates the different potential psychological mediators and organizes them within a hierarchical multilevel theoretical framework.

**Figure 3:**
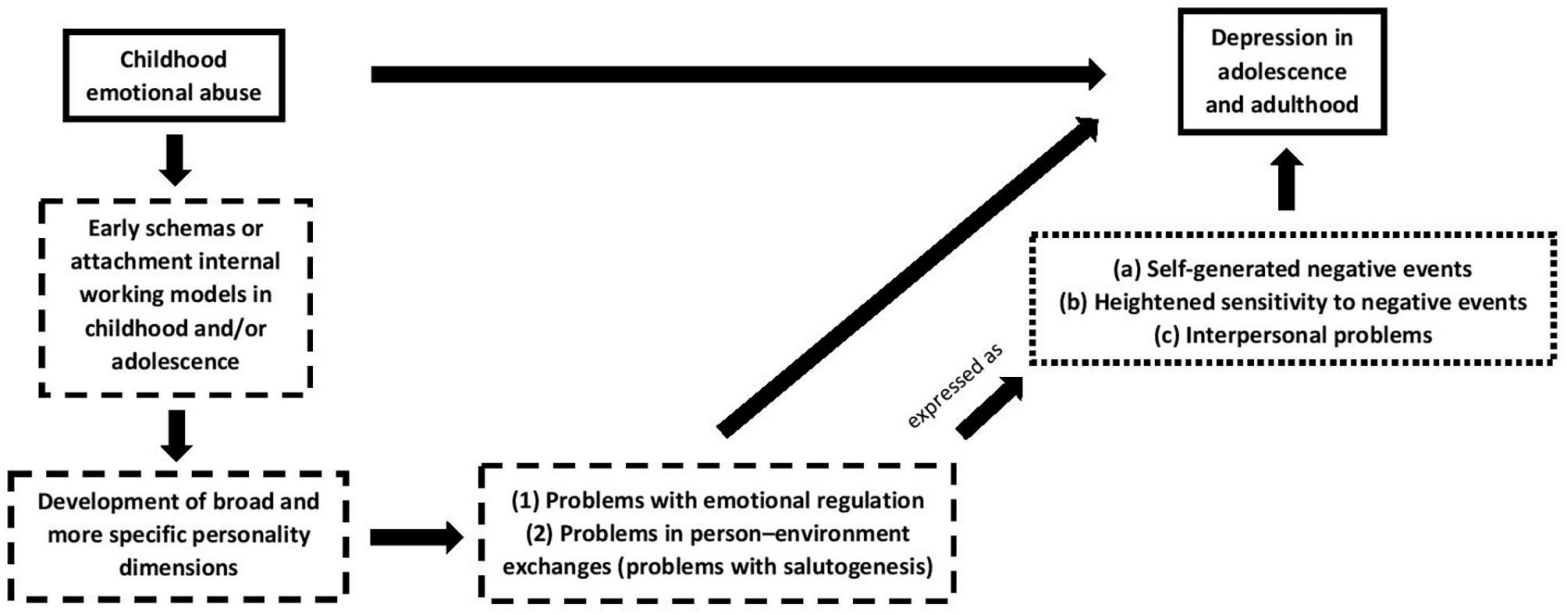
A multilevel integrative model of the relationship between childhood emotional abuse and adult depression.

In this model, childhood emotional abuse and various psychological mediators are considered to have direct and indirect effects on symptoms of depression (see Figure 3). Specifically, childhood emotional abuse is hypothesized to affect early schemas or attachment internal working models in childhood and/or adolescence, which in turn influence the development of broad and more specific personality dimensions, resulting in problems with emotional regulation and problems in person–environment exchanges (i.e., problems with salutogenesis), further expressed as (a) self-generated negative events, (b) heightened sensitivity to stressors, and (c) interpersonal problems in adolescence and adulthood.

In this integrated model, experiences of emotional abuse are thought to first affect early schemas or attachment internal working models in childhood and/or adolescence. Studies included in the current review investigated the role of maladaptive schemas in the relation between emotional abuse and depression based on Young and colleagues' schema theory (105). Four studies addressed the pathways of particular schema themes that mediated the emotional abuse–depression link, including vulnerability to harm, self-sacrifice, social isolation, defectiveness and shame, disconnection and rejection, impaired autonomy, loneliness, and submission. It has been suggested that early maladaptive schemas might originate from unmet childhood needs for secure attachment (139). However, only two studies (31, 51) included in our review assessed attachment styles as a potential mediator, finding that insecure attachment styles prospectively mediated the association between a childhood history of emotional maltreatment and depressive symptoms in young adults, but were not significantly correlated with childhood emotional abuse or current depression in a small sample of community-dwelling older adults.

In this integrative theoretical model, we consider that early schemas and attachment internal working models in childhood and adolescence give rise to the development of broad and more specific personality dimensions (i.e., character tendencies and cognitive and emotion-related factors), resulting in problems with emotional regulation and problems in person–environment exchanges (problems with salutogenesis). The schemas of vulnerability to harm and self-sacrifice appeared as mediators in the emotional abuse–depression link in our review. The schema of vulnerability to harm highlights fear and helplessness about the future as a lasting sequel of emotional abuse, which might consolidate into a more pervasive negative cognitive style (31). There was robust evidence for a cognitive vulnerability pathway linking childhood emotional abuse and later depression. Especially consistent evidence was found for hopelessness mediating the emotional abuse–depression link (30, 65, 67). These findings fit with the hopelessness theory of depression developed by Abramson et al. (111), which postulates that chronic experiences of emotional abuse across different situations will induce hopelessness and lead to the development of an internal, stable, and global inferential style; this type of inference is generalized to other negative events, which accounts for subsequent increases in depressive symptoms. Gibb et al. (66) found that the relation between childhood emotional maltreatment and non-endogenous major depression was fully mediated by the presence vs. absence of negative cognitive styles; however, this was no longer the case when hopeless depression was controlled for. As such, it appeared that the etiological chain of the hopelessness theory accounted for only part of the emotional abuse–depression link. This assumption was also supported by Gibb et al. (67) who found that childhood emotional maltreatment led to negative attributions and inferences about specific experiences relating to emotional maltreatment, rather than cognitive vulnerability more generally.

In comparison, the schema of self-sacrifice refers to an excessive focus on others' desires and responses at the expense of one's own needs, so that individuals who are self-sacrificing typically believe that their own needs and emotions must be suppressed and inhibited (84). Hence, the schema of self-sacrifice might contribute to problems with emotional regulation as reflected in emotional inhibition and thought suppression, which are further demonstrated as experiential avoidance. Early maladaptive schemas refer not only to the content of one's thoughts but also to the repetitive and ruminative nature in which the person focuses on negative thoughts. Lumley and Harkness (89) found that tightly connected negative schema statements and loosely connected positive schema statements mediated the emotional abuse–depression link. This repetitive, ruminative nature of maladaptive schemas overlaps the cluster of emotion dysregulation in brooding rumination, consistent with the finding that brooding rumination was a mediator in the relation between early maladaptive schemas and symptoms of depression in adolescents (140). As such, the different clusters of mediators considered in the context of the present review can overlap significantly, and most probably interact with and reinforce each other.

Yet, Young et al. (105) have argued that early maladaptive schemas originate as the result of the interaction between early experiences and the child's temperament. The influence of emotional abuse on maladaptive schemas and cognitive vulnerabilities might be particularly strong when early experiences interact with specific temperament dimensions, such as high Neuroticism and low Extroversion (141). Consistent with this assumption, high Neuroticism, low Extroversion, and low Conscientiousness have not only been consistently linked to the onset of MDD (115) but have also been found to be a mediator in the emotional abuse–depression link (69). Calvete (62) examined temperament as a moderator in a 1-year follow-up to test the hypothesis that parental emotional abuse interacting with temperament predicts a worsening of maladaptive schemas. However, emotional abuse and temperament seemed not to be related in their study. Thus, a direction for future research is to clarify the mechanism through which specific temperamental diatheses interact with early emotional abuse in the ongoing development of specific schema themes, thereby resulting in vulnerability to depression.

Finally, problems with emotional regulation and problems in person–environment exchanges (problems with salutogenesis) are expressed as (a) self-generated negative events, (b) heightened sensitivity to stressors, and (c) interpersonal problems in adolescence and adulthood. Both Hankin (31) and Uhrlass and Gibb (82) reported that stress associated with negative life events served as the mechanism linking childhood emotional abuse to current depression symptoms. Regarding stressful negative events, it is important to distinguish between (a) occurrence of life stress, which may be in part self-generated, and (b) subjective perception of life events. Hankin (31) measured a prospective increase in the number of negative life events that typically occur for young adults, from school/achievement to interpersonal/romantic difficulties. Uhrlass and Gibb (82) focused on hassles rather than major negative life events (e.g., deaths or loss of job) in their study, and levels of negative life events were calculated by summing the number of hassles endorsed rather than the subjective impact ratings. Thus, both studies fall into the category of occurrence of life stress, and are consistent with Hammen's model where childhood emotional abuse may set the stage for self-generated negative events and depressive symptoms, as well as the elaborated cognitive vulnerability–transactional stress model of depression (142). Hankin (31) claimed that childhood emotional maltreatment does not appear to serve merely as an additional risk factor for depression; rather, it may contribute to the onset of an ongoing cycle of negative events and depression in adulthood.

In respect of the subjective perception of life events, individuals with a history of childhood emotional abuse are at greater risk of developing depression through the proximal factor of a negative cognitive style for depression, which noticeably overlaps the cluster of cognitive-personality variables. This subjective perception of life events is highly intertwined with heightened sensitivity to stressors. Post's (143) stress sensitization hypothesis proposes that a history of childhood maltreatment lowers the threshold of stress necessary to trigger depression onset, so that individuals with a history of childhood maltreatment are more reactive and responsive to stressors (144–146). In addition, parental emotional abuse is likely to increase one's negative appraisal of stressful life events, and this relationship might be mediated by affective temperaments (147). Although Hayashi et al. (69) found no correlation between negative life events and personality, Monroe and Harkness (145) suggested that people with a characteristically negative style of interpersonal interactions tend to have more stressful life events. Future research should examine whether temperament interacts with stressful negative life events when integrated into the same structural model to help elucidate the common and specific etiological factors for depression.

With regard to problems with emotional regulation being manifested as interpersonal problems in adolescence and adulthood, Barthel et al. (148) have argued that emotion regulation is directly linked with one's ability to effectively navigate the social world. Engagement in emotion regulation often occurs interpersonally with trusted others helping to regulate one's emotions (148). One study included in our review, by Christ et al. (32), identified two specific domains of interpersonal problems—cold/distant and domineering/controlling—as particularly important in explaining the emotional abuse–depression link. Children with a history of emotional abuse are likely to develop negative internal working models of the self and others as well as maladaptive schemas that interfere with social functioning as the child matures, leading to experiential avoidance and a lack of trust in others (149). This avoidance, as an emotion regulation strategy, in interpersonal relationships and social situations can be seen as a cold/distant interpersonal style. By contrast, the examination of a domineering/controlling interpersonal style in depression has received inconsistent results (125, 127). In addition to cold/distant and domineering/controlling interpersonal styles, other interpersonal problems, such as self-sacrificing, non-assertive, intrusive/needy, and overly accommodating, also theoretically relate to the emotional abuse–depression link. However, Sachs-Ericsson et al. (79) found no evidence of a dependent interpersonal style as a mediator in this link, although the dependent characteristic reflects extreme distress in relation to a strong need for approval and has been posited as a vulnerability marker for depression. Future studies are needed to examine which specific interpersonal styles stem from childhood emotional abuse, and to what extent these types of interpersonal problems may be associated with a greater risk of depression.

### Directions for Future Research

Our findings support several potential psychological routes with relative consistency to adolescent and adult depression following a history of childhood emotional abuse (e.g., early maladaptive schemas, hopelessness, negative cognitive styles, brooding rumination, and overall emotion dysregulation). Further investigation is recommended to replicate the findings of those mediators that have been examined only a few times (e.g., personality traits, interpersonal styles). It is highly likely that considerable overlap exists between various mediating constructs and that several mediators may represent manifestations of other underlying processes (e.g., the important role of cognitive processes underlying the regulation of emotion). There is a lack of clarity regarding the extent to which these processes are relatively independent from each other, as well as their relative contribution to explaining risk for depression in those who have experienced childhood emotional abuse. Future research is needed to (a) compare the effect size of different mediators, (b) explore the interrelation between various mediators, (c) determine the degree of overlap, to disentangle the independent contribution of these different processes, and (d) elucidate whether certain types of experiences are particularly likely to trigger certain mechanisms. It is also possible that some of the psychological variables reviewed in the current study have a moderating role, as opposed to a mediating one, in the emotional abuse–depression link. For example, Hoppen and Chalder (24) found that romantic attachment avoidance and low self-esteem served as moderators, rather than mediators, in the relationship of childhood adversity with affective disorders. It is therefore unwarranted to assume that factors that do not appear to mediate the emotional abuse–depression link in this review do not play a role in the development of depression.

Current theories and evidence explaining the emotional abuse–depression link are largely built on isolated investigations based on researchers' interests and constrained by research funding and sample sizes, which may at times narrow findings at the expense of the bigger picture. To scrutinize whether psychological mediators function as a complex interrelated system in the emotional abuse–depression link, this review proposes a multilevel integrative model. Such an approach would help to move isolated focuses forward to empirical research based on a more comprehensive theoretical model that incorporates the different potential psychological mediators. This future work will require larger sample sizes, longitudinal designs, and more complex modeling techniques, allowing the robust appraisal of different pathways from childhood emotional abuse to subsequent depression.

Future research should also take resilience and protective factors into consideration. Not all emotionally maltreated children develop lifelong maladaptation. A focus on the individual differences between childhood emotional abuse victims can help us understand how some people overcome the emotional challenges of growing up in such environments. Studied protective factors for childhood maltreatment include attributional style (e.g., attribute abuse to external causes), early childhood secure attachment, and environmental factors, such as school and the presence of supportive relationships (150, 151). This line of research will help establish protective factors across the family system, community, school, and peer group to moderate the effects of parental emotional abuse.

Finally, future research should involve neurobiological processes related to identified psychological mechanisms in investigating the paths. Childhood adversity has been shown to be particularly detrimental, with long-lasting effects, during early stages of brain development (152). The causes of depression are most likely to be multifactorial, with interplay between genetic, neurobiological, psychological, and social factors. This line of future work will benefit from the integration of neurobiological and psychosocial approaches to illuminate the pathways that lead from childhood emotional abuse to depression in adolescence and adulthood.

### Limitations

Findings should be considered in light of some limitations. Based on our quality assessment, more than half of the included studies (*n* = 18) were judged to be at high risk for selection bias, 13 studies did not control for the influence of potentially relevant confounders, such as age, gender, ethnicity, and socioeconomic status, and, importantly, eight studies were rated as weak in terms of the relative strength of the statistical mediation analysis. Given that the relationship between childhood maltreatment and later affective disorders has been found to be dependent on the age of exposure (153, 154), doses (2, 155), and severity (20, 156), a level of caution is required in the interpretation of the relative contribution of the mediators examined in the studies receiving weak ratings. In addition, more than two-third of the eligible studies (*n* = 24) were cross-sectional, where the relationship between the mediators and depressive symptoms is hard to disentangle as mediation is an inherently longitudinal process. Given that the quality of the studies might be impaired by the bidirectional relationship between risk factors for depression and depressive symptoms, the mediators identified in the cross-sectional studies should be accepted with caution.

An outstanding potential moderator that needs to be taken into consideration is the age of exposure. Twenty-six studies used adult samples (including one study investigating older participants), of which 11 used young adults recruited from universities, and eight studies used adolescent samples. Childhood emotional abuse might be more strongly predictive of depression onset in adolescence than in adulthood (157, 158), and the depressogenic effect of childhood maltreatment has also been shown to be stronger in adolescence than in adulthood (159). Considerable research has pointed to the dramatic increase in interpersonal stressful events and heightened sensitivity to social situations that occurs during adolescence as significant contributors to depressive symptoms. Affective processing (160) and top-down processes involved in understanding and identifying emotions are still developing during adolescence (161). Adolescence is a developmental period where individuals often experience their first depressive episode (162). The point prevalence of MDD in adolescence ranges from 3 to 8%, with lifetime rates reaching 20% by the end of adolescence (163). La Rocque et al. (164) examined the moderating role of age in the relation of childhood maltreatment with sensitization to stressors that occurred just before episode onset. They found that adolescents (but not adults) with a history of maltreatment reported a lower level of severity of life events before episode onset than those without such a history; moreover, this relation was specific to emotional abuse, not physical or sexual abuse. As such, adolescents with a history of childhood emotional abuse may be more strongly sensitized to stressful life events that are crucial to triggering depression than adults with a similar history. Moreover, given that childhood maltreatment is closer in time to depression onset for adolescents who may still be living in an environment characterized by family discord and parental cruelty than for adults (165), childhood emotional abuse is likely to have a stronger etiological relation to depression onset in adolescence than in adulthood.

Notably, a majority of studies have samples composed of participants who are primarily Caucasian, women, university students, high-risk groups, low-income groups, or some combination thereof. One-third of included studies (*n* = 11) were conducted with young adult participants recruited from universities, who represent a fairly high-functioning sample with relatively homogeneous backgrounds. As such, the findings may be limited in generalizability to other populations. Nonetheless, well-educated young adult women have been shown to be an important high-risk group for developing depression (166). Not only are women twice as likely as men to experience MDD (167); a recent survey across eight countries demonstrated depressive disorder to be the most common mental disorder in first-year university students, with a 12-months prevalence of 18.5% (168). In addition, only a few mediators in eligible studies (e.g., early maladaptive schemas) have been examined in both clinical samples and non-clinical community samples. It will be important to examine whether the current findings of various mediators are replicated in both clinical and general samples and in more ethnically diverse samples.

Another limitation that has been repeatedly pointed out in the literature is the reliance on retrospective recall of childhood emotional abuse. There is a possibility of age and memory biases during affective episodes related to childhood maltreatment. As mentioned, some studies have reported that childhood maltreatment affected adolescents and young adults more strongly than middle-aged and older adults (164). However, some researchers found that childhood maltreatment had certain effects on older adults (169). Recent studies suggest the recall and mood effect can be negligible and retrospective reports can be highly consistent with prospective designs (170–172). In addition, as studies using a cross-sectional design do not allow any firm conclusions to be made regarding the direction of the present relationship between childhood emotional abuse, potential mediating factors, and later depression, in order to arrive at more solid ground it would be necessary for each potential mediator to be replicated in a longitudinal design.

There were also some limitations to the review itself. While our search strategy was sensitive and incorporated the screening of references and citations of eligible studies, included articles were restricted to those published in peer-reviewed English-language journals, which may have resulted in relevant evidence being overlooked. The exclusion of unpublished reports, including master's and doctoral theses, may impact on the current findings, as published sources may overrepresent outcomes where significant mediation via psychological processes is observed. However, our search strategy did not include the terms “mediator” or “mechanism” and therefore we were more likely to retrieve studies reporting diverse, rather than only positive, findings. Future evidence syntheses may include more thorough examination of gray literature or estimate the extent to which publication bias and other selection biases might affect the findings of the review. Despite these limitations, the current review represents an initial step in building a comprehensive multilevel theoretical framework to guide future more integrative research efforts in this field.

### Clinical Implications

Childhood maltreatment has been linked to treatment resistance in affective disorders (9, 173, 174) as well as greater functional impairment (175). Some researchers have claimed that childhood maltreatment-related affective disorders should be considered a clinically distinct subtype (176). Our results are in line with numerous studies arguing that early abusive experiences should be routinely assessed in clinical practice, and it is important for clinicians to recognize and assess any core dysfunctional beliefs. In comparison with other more “tangible” forms of maltreatment, a history of emotional abuse may be more elusive and subtle to detect in intervention. The very nature of emotional abuse contributes it the possibility that it may “hide in plain sight” from practitioners and help to explain the fact that few interventions exist that explicitly target a history of parental emotional abuse (43). Our findings represent potentially valuable intervention targets that clinicians should consider while developing prevention and treatment plans for victims of childhood emotional abuse who have distressing symptoms of depression. As these mediators are very likely to function as a complex interrelated system, clinical work should include work on identified pathways, with the awareness that addressing each one may also positively impact on the others.

Cognitive and emotion-related factors, sensitivity to stressors, emotion dysregulation, and interpersonal style are often explicitly targeted in therapeutic and preventive interventions that are already recommended for depression, such as cognitive-behavioral therapy, interpersonal psychotherapy, emotion-focused therapy, acceptance-based psychotherapeutic interventions, such as dialectical behavior therapy and acceptance and commitment therapy, as well as psychodynamic and mindfulness-based approaches. Schema therapy, which includes key strategies to modify early maladaptive schemas, such as emotional imagery, interpersonal techniques, cognitive restructuring, and self-empowerment exercises, would be helpful for victims of emotional abuse with chronic dysfunctional schemas. This review highlights the importance of personalized therapy plans, as well as preventive and public health interventions aimed at individuals with a history of childhood emotional abuse.

## Conclusions

The current systematic review demonstrates several potential mechanisms that might be involved in the relation between childhood emotional abuse and adolescent and adult depression. These psychological mediators fall into five clusters: (1) early maladaptive schemas, (2) cognitive-personality variables, (3) emotion dysregulation, (4) interpersonal styles, and (5) stressful negative events. However, the choice of examined variables to date has been limited and selective, suggesting that the conceptual framework guiding research in this field needs an integrative developmental cascade approach. Potentially significant elements of the emotional abuse–depression link may remain invisible as relevant variables have not been sufficiently examined. For example, we found no study examining either guilt or anger/aggression as a mediator, although they are two central concepts in the psychodynamic understanding of depression. We also hope to see more research on resilience and protective factors that might buffer children who are at risk for emotional and behavioral maladjustments. Most importantly, our postulated multilevel integrative model aims to inspire future research that directly addresses a coherent and integrated developmental theory or model in the emotional abuse–depression link. Ultimately, we hope to see a shift from isolated focuses in this area toward empirical research based on a more comprehensive theoretical model that incorporates the different potential psychological mediators and organizes them within a hierarchical multilevel theoretical framework.

## Data Availability Statement

The original contributions presented in the study are included in the article/supplementary material, further inquiries can be directed to the corresponding author/s.

## Author Contributions

EL conducted the systematic search and data extraction. Uncertainties during this period were resolved through discussions among the authors. The initial writing up was performed by EL under the supervision of PL and NM. All authors contributed to the manuscript writing and further elaboration and approved the final manuscript.

## Conflict of Interest

The authors declare that the research was conducted in the absence of any commercial or financial relationships that could be construed as a potential conflict of interest.
